# Preparation and Characterization of the Functional Properties of Synthetic Aggregates from Silico-Manganese Slag

**DOI:** 10.3390/ma14237303

**Published:** 2021-11-29

**Authors:** Zhibing Xing, Fenglan Han, Jiuliang Tian, Zhichao Xu, Jiaqi Wang, Tengteng Liu, Bin Zheng, Jiahe Huang

**Affiliations:** 1 School of Material Science and Engineering, North Minzu University, Yinchuan 750021, China; 20197201@stu.nun.edu.cn (Z.X.); 20190313@stu.nun.edu.cn (J.T.); 20180240@stu.nun.edu.cn (Z.X.); 20207320@stu.nun.edu.cn (J.W.); 20207321@stu.nun.edu.cn (T.L.); 20207330@stu.nun.edu.cn (B.Z.); 20197320@stu.nun.edu.cn (J.H.); 2 International Scientific & Technological Cooperation Base of Industrial Waste Recycling and Advanced Materials, Yinchuan 750021, China

**Keywords:** aggregate, bulk crushing strength, curing temperature

## Abstract

A large number of natural aggregates are used in the field of construction materials, resulting in the exhaustion of natural aggregates. Therefore, looking for an alternative will slow down the consumption of natural aggregates. The sintering method not only consumes a lot of energy to prepare aggregates but also produces a lot of pollutants. In this study, silico-manganese (SM) slag was dried, ground into powder, and used as raw material. Solid and liquid alkaline activator methods were used to prepare SM slag non-burning aggregate (SMNA) by the cold bonding method. The effects of grinding time, amounts of solid and liquid alkaline activators, curing temperature, and the amount of added fly ash on aggregate properties were investigated. The aggregate microstructure was characterized by XRD, SEM, and FTIR methods, and the toxic leaching analysis of aggregate was performed. The results showed that with a fixed amount of liquid activator (16.2% wt.) and solid activator (15% wt.) and fly ash (20% wt.), respectively, and curing was performed at room temperature, the aggregate properties were optimal: the bulk density of 1236.6–1476.9 kg/m^3^ and the water absorption lower than 4.9–5.5%. The apparent density was 1973.1–2281.6 kg/m^3^, and the bulk crushing strength was 24.7–27.9 MPa. The XRD, SEM, and FTIR results indicated that amorphous gel could be formed from SM under an alkaline activator, improving the aggregate strength. The results of toxic leaching showed that the aggregate prepared from SM exhibited environmentally friendly characteristics. The SMNA was obtained via the simple and low-energy consumption production process, paving the new way toward large-scale utilization of SM.

## 1. Introduction

Concrete is an indispensable building material in construction engineering. Aggregate is abundant in the concrete mix [1], accounting for about 70% wt. [2]. Extensive use of natural aggregate yields a shortage of high-quality aggregate and an increase in aggregate cost [3]. At the same time, it will have a serious impact on the ecological environment [4]. At present, it has been reported that industrial solid wastes such as fly ash (FA) [5], ground granulated blast furnace slag (GGBFS) [6], and steel slag [7] are used to prepare aggregates to replace some natural aggregates in concrete. It can not only slow down the mining of natural aggregates but also reduce environmental pollution. Therefore, using solid waste in artificial aggregate instead of natural aggregate is an effective way to reduce the consumption of non-renewable resources.

SM slag is a by-product of SM alloy smelting. According to statistical data from 2018, China’s SM alloy production exceeded 6.6 million tons, accounting for 20% of the total production of ferroalloy, among which Inner Mongolia, Ningxia, and Guangxi are areas that produce the most of SM alloy, representing 72% of the national output [8].

However, the production of 1 tone of SM alloy yields 1.2 tons of SM slag [9], continuously increasing the SM slag amount. Most companies that produce SM alloy do not further process the generated SM slag but discharge and accumulate it in the open air arbitrarily, yielding many harmful substances into the environment [10]. At present, Patil and Pande [11] have analyzed the properties of SM slag and found that its high wear resistance and freeze-thaw resistance, and high strength when used as a concrete admixture in the later stage, so the SM was widely used in roadbed materials. Li Wenbin et al. [12] used a mechanical shaft kiln to make cement clinker after grinding SM slag and nickel slag, and the utilization rate of the SM slag reached 35%. Allahverdi A. et al. [13] reported that water-quenched SM slag could replace 35% of Portland cement without volume instability. Zhang Dianyuan et al. [14] prepared an SM slag-based composite admixture with SM slag, limestone, and slag as raw materials under the action of grinding aids, meeting the requirements of S95 slag powder in GB/T 18046-2008. Frias M. et al. [15] showed that adding 15% of SM slag into Portland cement effectively improved the seawater erosion resistance of concrete, suitable for the concrete admixture of offshore buildings. Ting M. et al. [16] used silico-manganese slag, seawater, and sea sand as raw materials to prepare concrete and evaluated the durability of the concrete. The results show that the use of sea sand can effectively improve the durability of concrete and has potential industrial application value. Due to the limitations of concrete products, the utilization of SM slag in the above research is still rather low.

In producing low-cost building materials, cold bonding is considered a stable technology for waste recycling [17] by turning different kinds and large quantities of waste into valuable products. Compared with sintering, cold bonding granulation requires low energy consumption and yields little pollution [18].

Therefore, many researchers have recently prepared aggregates by using cold bonding methods of different wastes [19,20,21,22,23,24,25,26,27,28,29,30]. However, there are only a few reports on the preparation of non-burning aggregate from SM.

This research mainly opens up new ideas for the application of silicomanganese slag in artificial aggregates and manufactures substitutes for natural aggregates. In this paper, two alkaline activator methods with solid and liquid activators were used to prepare SMNA by a cold bonding method. The bulk density, cylinder compressive strength, water absorption, and apparent density of the SMNA were tested and analyzed, and the aggregate microstructure was characterized by XRD, SEM, and FTIR methods, and the safety of the SMNA was evaluated. Finally, the novel SMNA can effectively alleviate the demand for natural aggregate in the construction field, paving the new way for the large-scale application of SM slag. This technology is of great significance to solve the problem of massive accumulation of SM slag. It is also in line with the concept of green building development. It is also significant to protect the limited natural building aggregate resources and reduce the over-exploitation of stone mountains.

## 2. Experiment

### 2.1. Experimental Material

**Figure 1:**
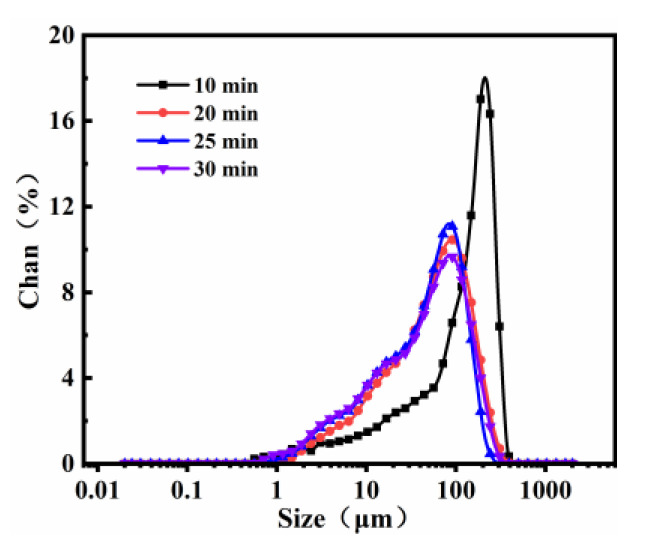
SM particle size distribution under different grinding times.

Dark green SM slag was supplied from the water quenching granular slag of an SM alloy enterprise in Ningxia. The SM slag was ground for 10, 20, 25, and 30 min using a sealed sample preparation mill. The D_50_ values of the SM slag were 121.2, 63.44, 54.06, and 53.43 µm, respectively, as determined using Mastersizer 2000 laser particle size analysis. The particle size distribution diagram for different grinding times is shown in Figure 1. The D_50_ values were determined for the SM slag sieved through a 60-mesh sieve after grinding. Fly ash (FA) with a D_50_ value of 15.97 µm was obtained from a power plant in Ningxia, Yinchuan, China.

**Figure 2:**
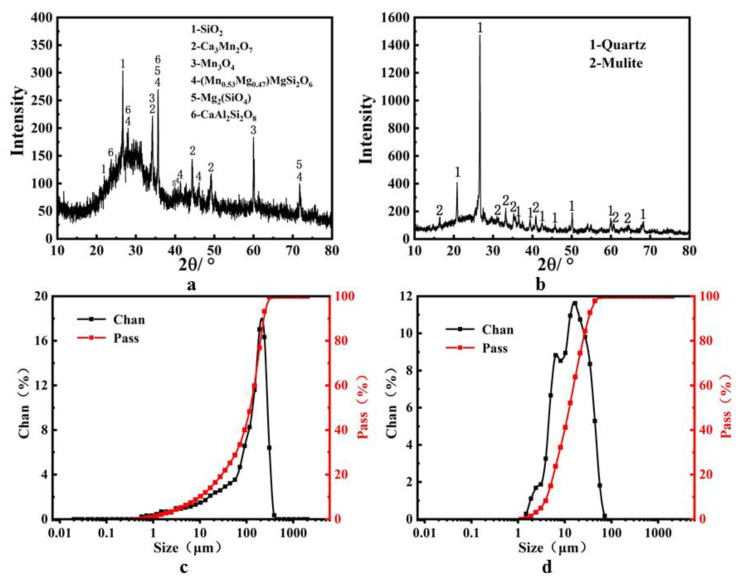
(**a**) XRD patterns of the SM; (**b**) XRD patterns of the FA; (**c**) particle size distribution of SM; (**d**) particle size distribution of FA.

**Figure 3:**
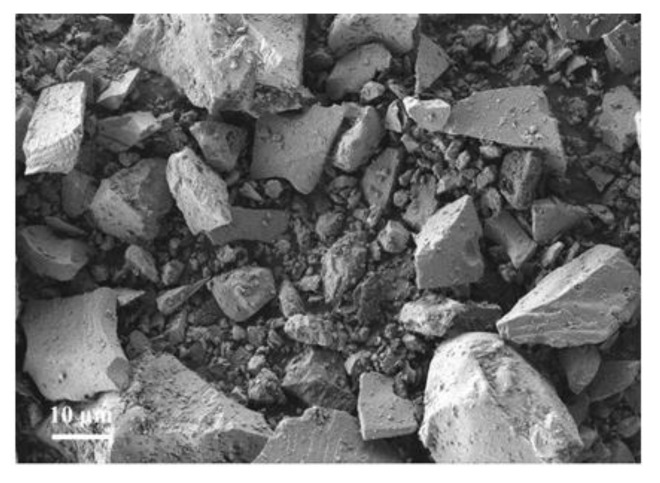
SEM images of SM slag.

The liquid activator consisted of the following components: a mixture of NaOH, sodium silicate, and water in proportion, where NaOH was analytically pure, sodium silicate was of the industrial-grade, module 2.7; the solid activator consisted of the following components: solid sodium silicate, module 2.0, a 100-mesh screening rate up to 98.9%. Table 1 shows the chemical composition of SM slag and FA. The XRD pattern and particle size distribution of SM slag and fly ash are shown in Figure 2. The SEM image of SM slag is shown in Figure 3.

Figure 2a shows the main physical phases in the water-quenched SM slag: quartz (SiO_2_), direct manganese (Mn_0.53_Mg_0.47_) MgSi_2_O_6_, magnesium olivine (Mg_2_SiO_4_), calcium feldspar (CaAl_2_Si_2_O_8_), Mn_3_O_4_, and Ca_3_Mn_2_O_7_. According to Figure 2b, the main physical phases in fly ash are quartz (SiO_2_) and mullite (3Al_2_O_3_·2SiO_2_). According to Figure 2c,d the SM slag particle size distribution is narrow, while FA exhibits a broad particle size distribution.

Figure 3 shows many massive particles in SM, having polygonal and irregular shapes, uneven particle size distribution, and many dense particles on the surface.

### 2.2. Experimental Scheme

Table 2, Table 3 and Table 4 show the preparation scheme of SMNA.

### 2.3. Physical and Mechanical Characterisation

1 h water absorption, apparent density, and bulk density of aggregate. According to GB/T 17431.2-2010 for determination.

The aggregate with a curing time of 3 days and a particle size of 4.75–9.5 mm was dried for 2 h in a drying oven at 105 °C to determine the bulk density and then loaded into a 500 mL measuring cylinder to test its quality. The average value was taken for two tests.

(1) $$\mathrm{Packing}\;\mathrm{density}\;\rho_{1}=\frac{m_{1}}{V}\times 1000$$

where $\rho_{1}$ represents the bulk density in kg/m^3^, $m_{1}$ represents the quality after drying in g, and *V* stands for the volume of 500 mL cylinder in milliliters.

The 1 h-water absorption rate and apparent density of aggregate were tested. The aggregate with a curing time of 3 days and a particle size of <4.75 mm or 9.5–15 mm was dried in a drying oven at 105 °C for 2 h and then tested. About 75 g were weighed each time, and the average value was taken for three tests.

Water absorption test: the dried aggregate was added into a 250 mL beaker and soaked in water for 1 h; afterward, it was placed in a sieve with a diameter of 2.36 mm to filter water, rolled back and forth with a wrung towel for ten times to wipe the moisture on the aggregate surface, and finally the aggregate quality was weighed, and the water absorption rate was calculated.

Apparent density test: in accordance with the previous steps of the water absorption test, the dried aggregate was finally poured into a 250 mL measuring cylinder filled with 130 mL water. The water surface scale was read to calculate the apparent density.

(2) $$\mathrm{apparent}\;\mathrm{density}\;\rho_{2}=\frac{m_{1}}{250-130}\times 1000$$

(3) $$1\;h-\mathrm{water}\;\mathrm{absorption}\;\omega_{1}=\frac{m_{2}-m_{1}}{m_{1}}\times 100$$

where $\omega_{1}$ represents the water absorption rate in%, $\rho_{2}$ is the apparent density in kg/m^3^, $m_{1}$ is the quality after drying in g, and $m_{2}$ is the mass after water absorption in g.

**Figure 4:**
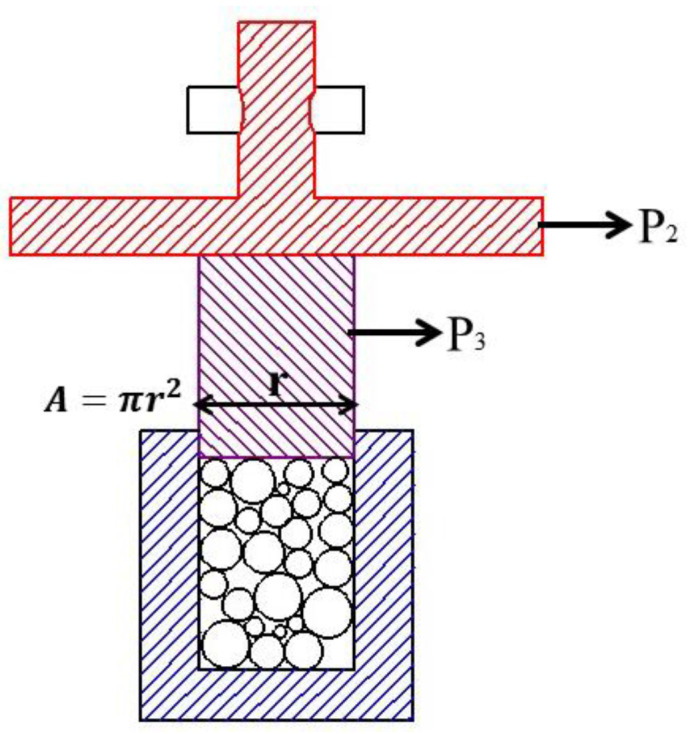
Schematic diagram of bulk compressive strength.

As shown in Figure 4, The compressive strength test of the aggregate cured for 3 and 28 days was performed according to GB/T 17431.2-2010. Firstly, the aggregate samples with a particle size of 4.75–9.5 mm were dried and loaded into a cylindric metal mold with an inner diameter of 50 mm. Then, the cylindric metal plug was loaded into the mold and placed on a microcomputer-controlled electrohydraulic servo universal testing machine (CMT5305), and pressed to 20 mm at a speed of 300 N/s. About 250 g was loaded into the pressure cylinder each time, and the average value was calculated three times.

(4) $$\mathrm{bulk}\;\mathrm{crushing}\;\mathrm{strength}:\;f=\frac{P_{1}+P_{2}+P_{3}}{A}$$

where f is the bulk crushing strength in MPa, $P_{1}$ is Press display in N;$\;P_{2}$ is the pressure plate quality in N;$\;P_{3}$ is the quality of the bearing rod in N; A is the bearing area in mm^2^.

The single-ball strength of FA/SM slag aggregate at different curing times was tested using a compression and flexion testing machine (YAW-300C) at a pressing rate of 1 mm/min until the particles broke. Eight aggregate samples with the same particle size and complete morphology were selected for testing each time, and the final results were given as average values.

(5) $$\mathrm{individual}\;\mathrm{pellet}\;\mathrm{strength}:\;\sigma =\frac{2.8F}{100\pi D^{2}}$$

where σ is the compressive strength of a single-particle aggregate in MPa; F is the load at the aggregate failure in N; D is the test diameter of aggregate in cm.

Elemental analysis was carried out on an X-ray fluorescence (XRF) analyzer (ZSX Primus II, Rigaku, Tokyo, Japan).

After the compressive strength test was completed, the crushed samples were soaked in anhydrous ethanol for 3 days. Before microstructural testing, they were dried for 12 h in an electric blast drying oven (101-3AB) at 105 °C before microstructural testing. After aging for 28 days, the crushed samples were ground into powder and analyzed by XRD and FTIR after sifting through a 200-mesh sieve. The fracture surface of the crushed sample was sputtered and tested using field emission scanning electron microscopy (SIGMA 500, Carl Zeiss AG, Oberkochen, Germany).

XRD measurements were performed to identify the phase composition using a Shimadzu X-ray diffractometer (XRD-6000, Shimadzu, Kyoto, Japan). A Cu target was used as the emission source, and the working voltage was 40 kV, the tube current was 30 mA, the measuring angle was between 10 and 80° 2θ, and a scanning speed of 2°/min.

A Bio-Rad FTS-6000 spectrometer (Bio-Rad, Hercules, CA, America) was used for the FTIR analysis in the wavenumber range from 4000 to 400 cm^−1^. Next, 1 mg of the powder sample sieved through a 200-mesh sieve was mixed evenly with 200 mg of KBr and then pressed in a tablet using a tablet press.

We used the national standard solid waste leaching toxicity of nitric acid sulfate method (HJ-557-2009) to evaluate the impact of aggregate on the environment. During the testing process, leaching was performed in a liquid to solid ratio of 10 l/kg, oscillating for 18 h in a rotating oscillation device. The content of heavy metal elements in the leaching solution was analyzed by an inductively coupled plasma atomic emission spectrometer (ICP-7000, PerkinElmer, Hopkinton, MA, America). The filtration value of elements in the leaching solution was compared with the standard limit value according to the leaching toxicity hazardous waste identification standard (GB5085.3-2007).

## 3. Preparation Process of SM Slag Aggregate

### 3.1. Process Flow of SM Granulation

**Figure 5:**
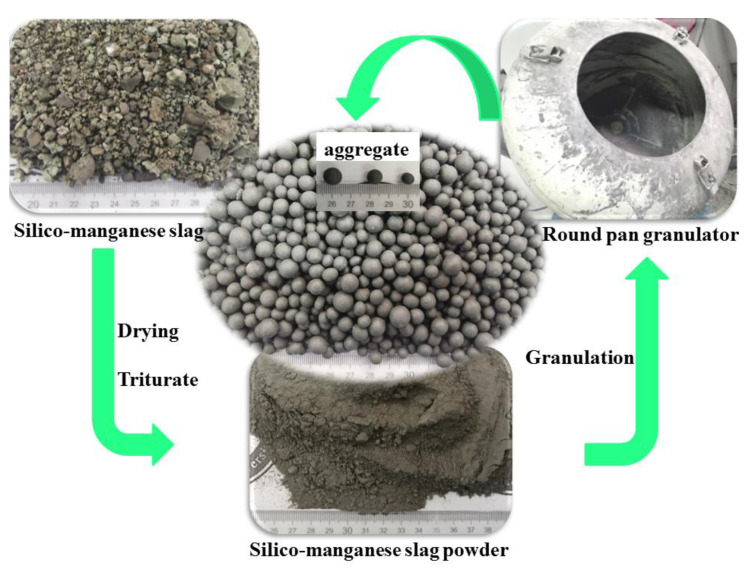
Flow chart of the granulation process.

**Figure 6:**
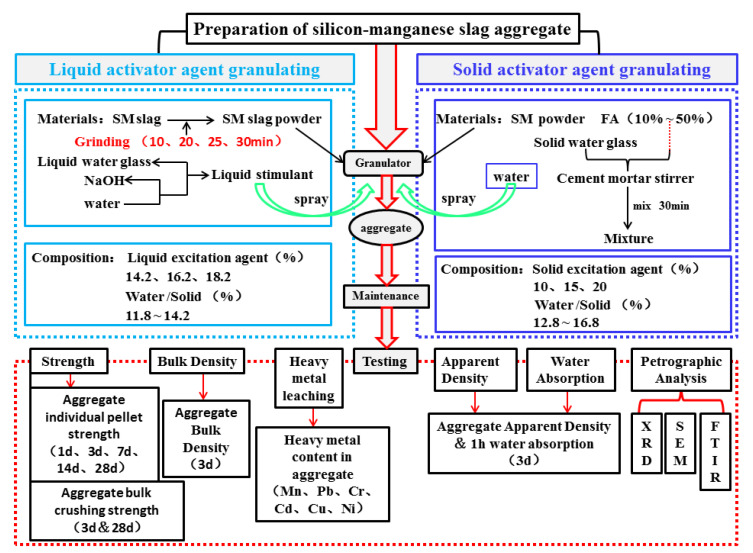
Preparation diagram of SMNA.

In this study, we used two granulation processes: the liquid activator granulation process and the solid activator granulation process. Figure 5 shows the granulation process flow chart, and Figure 6 illustrates the preparation diagram of SM slag aggregate.

#### 3.1.1. Liquid Activator Granulation Process

**Figure 7:**
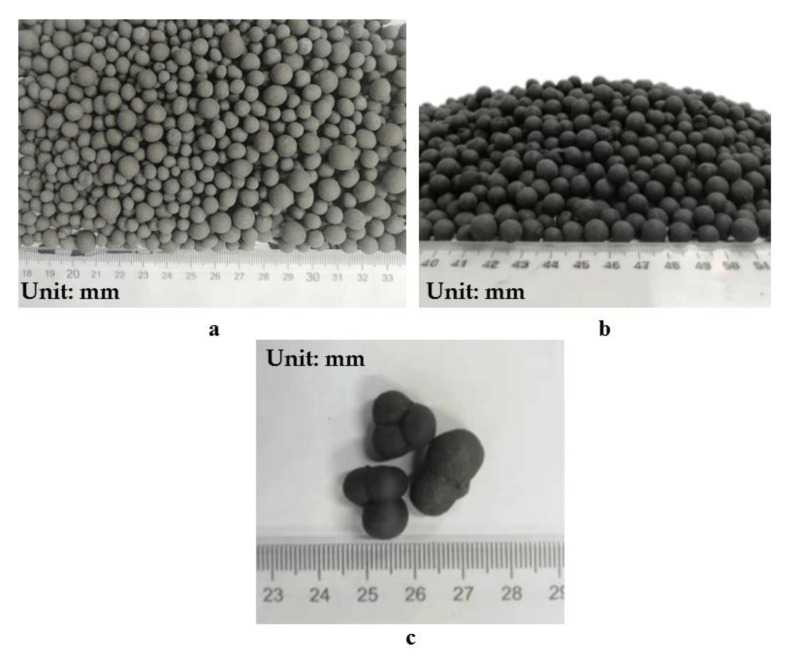
(**a**) Liquid activator used to prepare SMNA; (**b**) solid activator used to prepare SMNA; (**c**) bonding of pelletized graded broken aggregate.

Firstly, the granulated SM was dried and ground to a 60-mesh sieve. The prepared alkali activator mixture was stirred on a magnetic stirrer for 20 min and then poured into a sprayer. The inclination angle and speed of the granulator were adjusted to 50 ° and 12 r/min, respectively. Afterward, 3000 g of SM slag raw materials were added in several doses: the first 700 g of raw materials were put into the round pot granulator, and the spray granulation was started. When the particle size of the initial aggregate reached about 3–5 mm and the pelletizing time was about 6 min, the remaining material was slowly added for spray granulation until all 3000 g were added for about 20 min. After feeding, the aggregate surface was wet, and the granulator continued to roll until the aggregate surface became dry. At the end of the granulation process, the prepared aggregate was poured into the plate for laminating and curing. Figure 7a shows the liquid activator used to prepare SMNA.

#### 3.1.2. Solid Activator Granulation Process

Firstly, the granular SM was dried and ground to a 60-mesh sieve. Secondly, the solid sodium silicate and SM slag powder were mixed in the cement mortar mixer for 30 min to ensure uniform mixing of the powder and the solid sodium silicate. Afterward, the tilt angle of the granulator and the frequency of the frequency converter were adjusted to 50° and 12 r/min, respectively. In this experiment, the blended material was firstly divided into three parts, and the 700 g of the mixture was put into the round pot granulator to start spray granulation. When the initial aggregate particle size reached about 3–5 mm, and the pellet-forming time was about 15 min, the remaining material was slowly added for spray granulation until 300–500 g of the material remained. The aggregate surface was wet when the feeding was completed, and the remaining powder was added until the aggregate surface became dry. Subsequently, the granulation was finished, and the prepared aggregate was poured into the ceramic plate for laminating and curing. However, the adhesion between aggregates was stronger than for the liquid spray granulation when the ball-forming compaction was interrupted. The reason was that water continued to overflow the aggregate’s surface at the later stage because of the slow polymerization reaction between solid sodium silicate and powder, inducing strong agglomeration of the aggregate and forming multi-ball aggregates. Therefore, the water film on the aggregate’s surface should be avoided in this process. Figure 7b shows the solid activator used to prepare SMNA, and Figure 7c illustrates the bonding of pelletized graded broken aggregate.

### 3.2. Conservation Process of SMNA

The curing temperature has a very important influence on the performance of aggregate. The aggregate prepared by the liquid activator was set at 20, 30, 40, 50, and 60 °C for curing for 6 h, and then placed at room temperature for curing until age. The aggregate prepared by solid activator was set at room temperature, 40, and 60 °C for curing for 6 h, followed by curing to age at room temperature.

### 3.3. Pelletization Mechanism

The main forces affecting ball formation are natural and mechanical forces. The natural force is mainly based on capillary pressure, causing powder condensation and pellet formation. Moreover, the mechanical force is needed to induce the pellet’s collision, which is achieved in the pelleting equipment by mechanical movement of the powder via rolling and collision [31].

The pellet-forming process of aggregate is divided into three stages: material nucleation stage, stretching of the formed pellet material, and ball forming and compacting stage. These three stages are analyzed in detail. In the powder nucleation stage, the mixture of alkaline activator and water wet the material. This occurs due to the surface tension of the mixture of particles bound in small nucleated spherules since the particles adsorbed on the droplet’s surface and air droplets form a three-phase contact surface. Then, the granulation process begins, in which the atomization effect plays an important role in the granulation onset. In the pellet stretching stage, the amount of liquid phase between pellets gradually increases under the spraying action of spray, and the liquid is wrapped between small nucleated pellets. As the machine rolls, small nucleated pellets collide and extrude each other, which reduces the internal void of pellets and presents a solid-liquid-gas three-phase interface with low strength. Simultaneous spraying and rolling induce complete filling of the gap between the pellets with liquid. The bonding force makes the pellets more tightly bound, the amount of the gas phase is gradually reduced, the powder is sprinkled in the process, so the powder is evenly wrapped on the surface of the pellets. The particle size of the pellets gradually increases through the repeated wrapping. During the pellet-forming compaction stage, as the arranged balls continue to roll, the interior of the forming pellet gets more tightly pressed. The water inside the pellet spills over the surface and increases its plasticity until the surface becomes hard and the pellet-forming process is over.

## 4. Performance Test

### 4.1. Effect of the Grinding Time on SMNA

As can be seen from Figure 6, the bulk density of SMNA is in the range of 1192.0–1349.2 kg/m^3^, the apparent density is in the range of 2020.5–2140.5 kg/m^3^, and the water absorption is lower than 6.8%. When the grinding time is 30 min, the bulk crushing strength of SMNA reaches 22.1 MPa.

**Figure 8:**
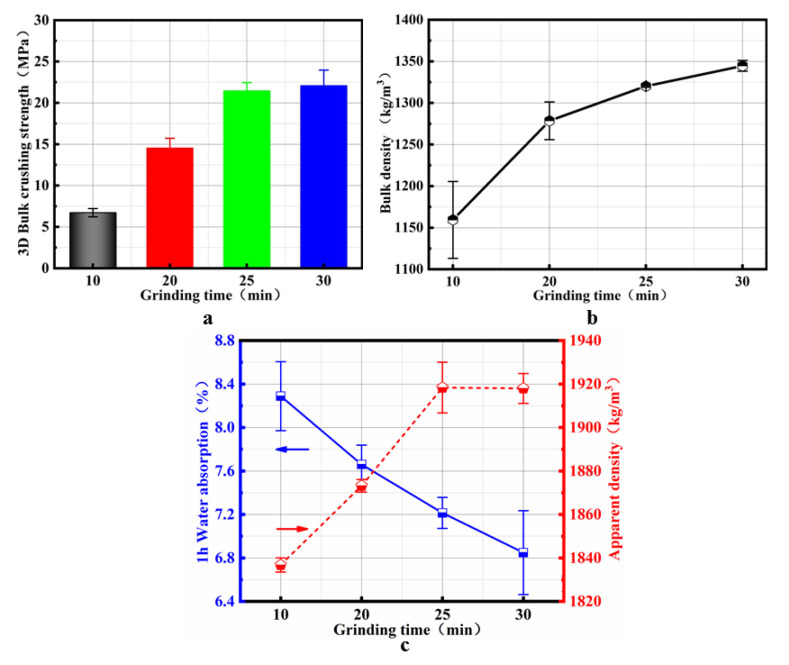
Effect of the grinding time on the properties of SMNA: (**a**) bulk crushing strength of SMNA; (**b**) bulk density of SMNA; (**c**) apparent density and water absorption of SMNA.

Figure 8a,b show that the bulk crushing strength and the bulk density of the aggregate cylinder increase gradually with the grinding time. The reason is that under mechanical force, the SM particles gradually refine, the specific surface area and the number of surface active sites increase, enabling full contact with the alkaline initiator polymerization reaction and increasing the strength. At the same time, a large number of refined particles can fill in the fine pores of the gel, making the gel structure more tightly bound. On the other hand, mechanical milling can distort the crystal structure of SM particles, reduce the crystallinity, and transform them into amorphous structures, increase the active components involved in the hydration reaction, and generate a lot of gel material.

As shown in Figure 8c, as the grinding time increases, the aggregate will generate more gel structures. a large amount of gel structure fills the internal pores of the aggregate, so that the water absorption rate of the aggregate gradually decreases.

### 4.2. Influence of the Curing Temperature on the Properties of SMNA

**Figure 9:**
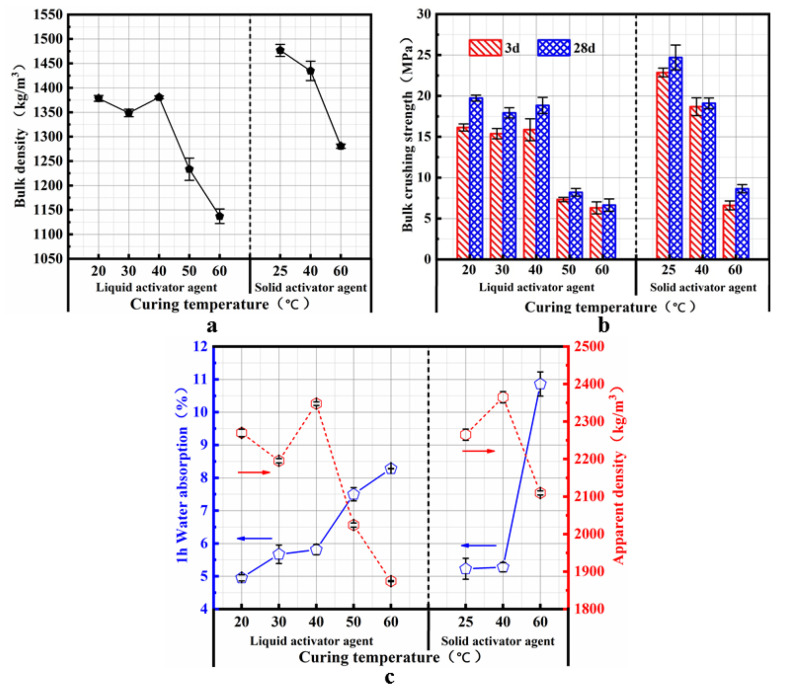
Influence of the curing temperature on the properties of SMNA: (**a**) bulk crushing strength of SMNA; (**b**) bulk density of SMNA; (**c**) apparent density and water absorption of SMNA.

As shown in Figure 9, when the curing temperature is between 20 and 40 °C, the strength after 3 days can reach 80% of the maximum strength after 28 days. The strength after 3 days at 50–60 °C can reach 70% of the maximum strength after 28 days. For the aggregate prepared by the solid activator, the strength after 3 days is 90% of the maximum strength after 28 days when cured at 25 °C. The SMNA exhibited early strength characteristics.

Curing temperature affects the cylinder compressive strength of the SM slag-free aggregate at 20–40 °C, but it decreases cylinder compressive strength at 50–60 °C. The interior of the aggregate will expand upon curing above 50 °C due to the conversion of the internal moisture into gas. The expansion during the curing process will cause certain thermal stress inside the aggregate, resulting in the aggregate’s internal structure expansion, causing structural cracks and pores in the aggregate. The structural expansion and the deterioration of the aggregate’s compactness decrease the bulk crushing strength of the cylinder. On the other hand, due to the very high curing temperature, the early moisture loss in the aggregate hinders the depolymerization of the active ingredients in the aggregate, so the ions move slowly in the liquid phase, affecting the degree of the polymerization reaction and causing the formation of the gel. Thus, it results in a decrease in the bulk crushing strength.

The water absorption rate gradually increases with the curing temperature; the bulk density and apparent density reflect the internal pore structure, and they can also indicate the internal pores of the aggregate when the curing temperature is higher than 50 °C. The accumulation and apparent density of the cylinder will decrease with the pressure strength, so the water absorption will increase. The change of the aggregate properties prepared by solid activators is consistent with the behavior of the aggregate prepared by liquid activators. The specific demonstration can be combined with the analysis of micro-morphology, and comprehensively, the aggregate’s performance is the best after the room temperature curing.

### 4.3. Influence of the Alkaline Activator on the Properties of SMNA

**Figure 10:**
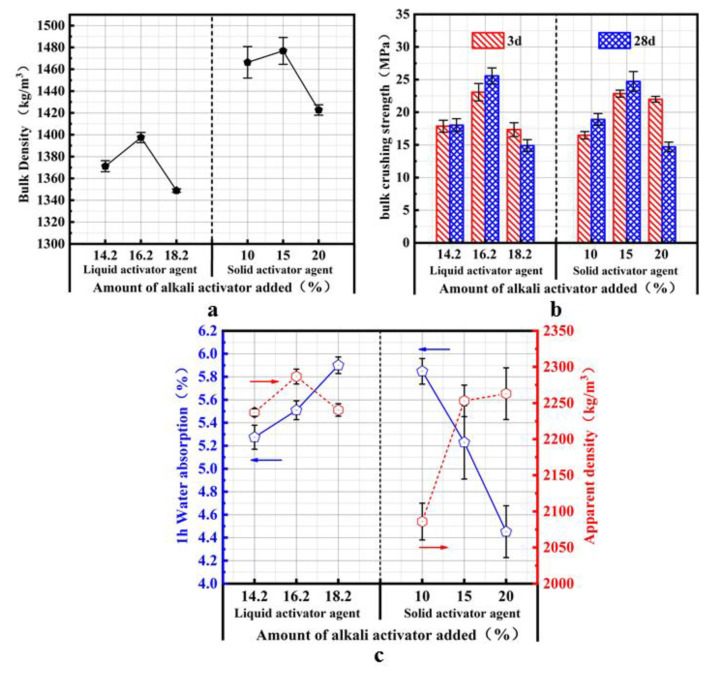
Influence of the alkaline activator addition amount on the properties of SMNA: (**a**) bulk crushing strength of SMNA; (**b**) bulk density of SMNA; (**c**) apparent density and water absorption of SMNA.

Figure 10a shows the aggregate prepared by liquid and solid activators, where the amount of the alkaline activator is 14.2, 16.2, 10, and 15%. The strength of the cylinder after 3 days reaches 68% of the maximum strength after 28 days, and the SM slag aggregate exhibits the characteristics of early strength. However, when the amount of added alkaline activator is 18.2 and 20%, the aggregate’s strength after 28 days decreases because a large amount of alkaline activator generates more gel in the aggregate, and the loss of water in the following curing process leads to aggregate’s shrinkage and cracking, so the strength decreases [32].

The bulk crushing strength of the non-burning aggregate cylinder of SM slag first increases and then decreases with the amount of liquid activator because the higher alkaline activator content yields an alkaline environment and silicon-oxygen tetrahedral groups in the aggregate, leading to the depolymerization of many vitreous structures in the SM slag and the fracture of Si-O and Al-O bonds. The dissolved oligomeric silicon-oxygen tetrahedra and the aluminum-oxygen unstable tetrahedral unstable enter the liquid phase, providing many precursors for the post-sequential polymerization reaction. With the progress of the polymerization reaction, morphous gels are formed, so the macroscopic performance exhibits a higher strength. Considering the addition of an alkaline activator, many Na^+^ ions exist in the system. They can partially cover the gel surface, resulting in the passivation phenomenon, which hinders further dissolution and release of active SiO_2_ and Al_2_O_3_ in the SM slag. It also suppresses the in-situ polymerization of SM slag, yielding a strength reduction. Therefore, the amount of liquid activator was chosen as 16.2%. With the addition of solid sodium silicate, the aggregate cylinder strength and liquid activator show the same behavior. When the amount of solid sodium silicate is 15%, the cylinder compressive strength after 3 and 28 days reaches the maximum value in this experiment. The main reason for this behavior is that the added solid sodium silicate dissolves a large amount of silicon and aluminum components in the SM slag, yielding a higher gel content and making the three-dimensional network structure more closely bound.

Figure 10b shows that the bulk density of aggregate prepared with the liquid activator is 1348.9–1397.5 kg/m^3^. However, the bulk density of aggregate prepared with the solid activator is 1422.7–466.4 kg/m^3^, and it initially increases and then decreases, consistent with the behavior of the cylinder compressive strength. This can also be explained by the higher amount of gel formed when an appropriate dose of the alkaline activator is added, filling the pores inside the aggregate and increasing the bulk density to the maximum at a dosage of alkaline activator agent of 16.2% and 15%.

Figure 10c shows that the apparent density of aggregate prepared with the solid activator is 2092.0–2276.9 kg/m^3^, while the apparent density of aggregate prepared with the liquid activator is 2235.7–2248.2 kg/m^3^. The water absorption for the two preparation methods is lower than 6%. The water absorption of the aggregate prepared with the liquid activator exhibits a descending trend, while it is opposite for the solid activator. The higher content of the Na_2_SiO_3_ liquid activator hinders the release of bubbles in aggregate and increases water absorption [33]. However, this also accelerates the reaction and yields rapid gel formation, preventing water molecules from entering the aggregate during the test process and increasing the water absorption trend.

### 4.4. Influence of the FA Amount on the Properties of SMNA

**Figure 11:**
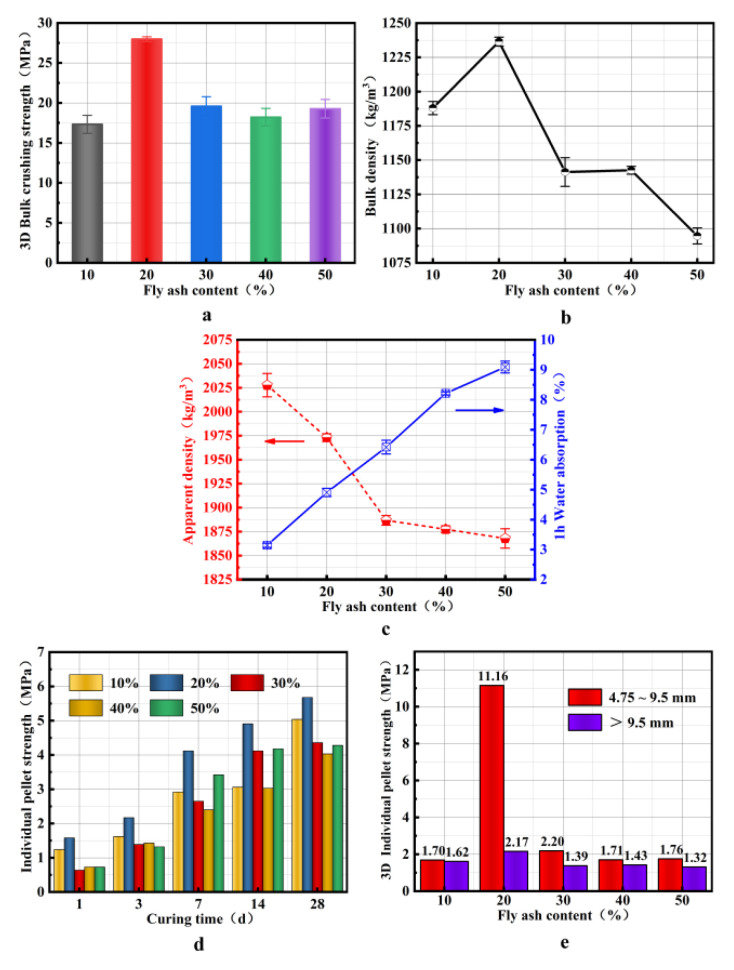
Influence of the added amount of FA on the properties of SMNA: (**a**) bulk crushing strength of SMNA; (**b**) bulk density of SMNA; (**c**) apparent density and water absorption of SMNA. (**d**) individual pellet strength of SMNA with different curing times; (**e**) individual pellet strength of SMNA with different particle sizes.

Figure 11a,b show that the bulk density first increases and then decreases with the FA amount, which is consistent with the behavior of bulk crushing strength. When the FA amount is 20%, the aggregate strength and bulk density reach the maximum. According to previous studies, the strength of geopolymer depends on the SiO_2_/Al_2_O_3_ ratio. An appropriate SiO_2_/Al_2_O_3_ ratio is required to obtain high strength geopolymer [34]. The appropriate SiO_2_/Al_2_O_3_ ratio is related to the selected material, especially the number of reactants in the selected material [21]. Therefore, according to Table 1, when the SiO_2_/Al_2_O_3_ mass ratio is 1.98, the aggregate strength is the highest because the appropriate SiO_2_/Al_2_O_3_ ratio can form a long-chain polymer with a more stable spatial structure [35]. Figure 11b shows that when the amount of fly ash added reaches 20%, the bulk density reaches the highest. The reason is that more gel is formed in the aggregate to fill the pores inside the aggregate, thereby maximizing the bulk density.

Figure 11c shows that increases with the FA addition, and the water absorption shows a gradual increase. The reason for such behavior is that the specific surface area of FA is greater than that of the SM slag, facilitating the water absorption, causing an increase in the water absorption rate of the aggregate. On the other hand, the high amount of FA may also extend the delay time, yielding many fine pores in the aggregate preparation process, which also leads to stronger water absorption.

Figure 11d shows that the individual pellet strength of aggregate exhibits the same behavior as the bulk crushing strength. As the curing time increases, the single-ball strength of aggregate gradually increases. When the FA content is 20%, the aggregate strength can reach 5.68 MPa after 28 days. When the curing time is from 3 to 7 days, there is the fastest increase of the aggregate strength, and when the amount of FA is 50%, the growth rate is 159%. However, with the extension of curing time from 7 to 14 days, the particle strength continues to increase rapidly. The rate of the particle strength growth decreases from 14 to 28 days. Figure 11e demonstrates that the aggregate strength increases with the decrease of particle size. This phenomenon is caused by the joint action of mechanical and capillary forces on particles with different sizes and size distributions in the disc and the uniformity of the powder and alkaline activator mixture [36]. Another reason is that water is more easily released from smaller particles during the pellet-forming and compactness granulation stages, yielding a denser matrix structure and higher strength of the smaller particles [21]. As the diameter increases, it decreases individual pellet strength of aggregate, which is consistent with the findings of Elisabeth [37]. However, when the FA content is 20%, the single-ball strength of 4.75–9.5 mm aggregate is 5.14 times higher than that of the aggregate > 9.5 mm because the randomness of the selected individual particles is large, leading to higher values.

### 4.5. Granulation Efficiency

**Figure 12:**
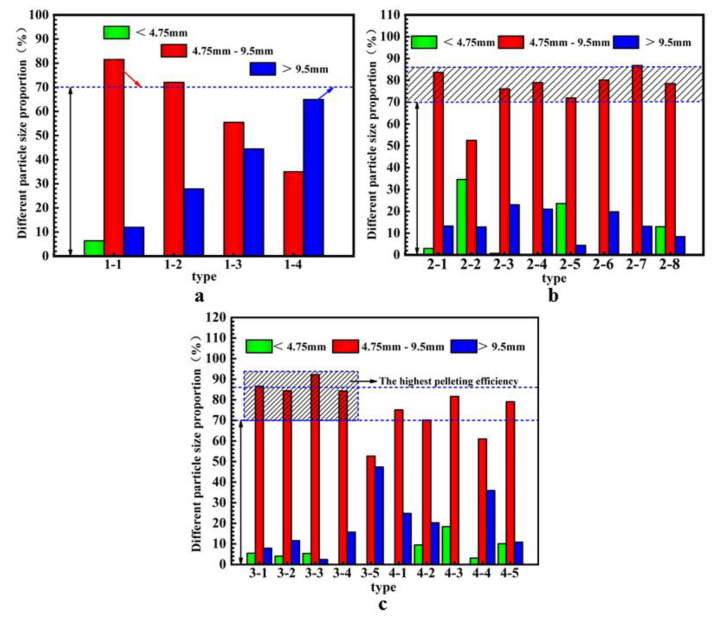
Effects of different variables on the granulation efficiency of different SMNA aggregates: (**a**) grinding time; (**b**) liquid activator; (**c**) solid activator.

The pelleting of SM slag is performed in a disc pelleter, and the maximum pelleting efficiency is provided by fixed speed and inclination angle. The pelleting efficiency is defined as the ratio of the produced 4.75–9.5 mm aggregate mass to the total prepared mass [30]. The aggregate particle size distribution prepared by using solid and liquid activators is shown in Figure 12.

The particle size range of the aggregates prepared by liquid and solid activators is 2.36–16 mm, exhibiting the adequate aggregate particle size distributions of the two preparation methods. Figure 12a shows that the aggregate particle size of 4.75–9.5 mm gradually decreases with the grinding time, while the particle size of the aggregate >9.5 mm gradually increases. The aggregate granulation efficiency also gradually decreases. More time and higher water content are needed to improve the pelleting efficiency in the pelleting process of SM slag with finer particles [38]. Since the water-cement ratio is consistent, the pellets quickly grow at the beginning of the granulation. In the pellet growth process, small particles gather into large pellets, leading to a decline in granulation efficiency. Figure 12b,c show that the aggregates prepared with solid and liquid activators have a 70–86% pelleting efficiency. In Figure 12c, the aggregate preparation using solid activator agents 3-1–3-4 exhibits the highest granulation efficiency, and among them, it is more than 90% for 3-3. These results can be explained by the better water atomization effect when using the solid activator agent in the aggregate preparation compared with the liquid activator agent, enabling the easy control of the initial particle size of the aggregate and improving the granulation efficiency.

## 5. Microstructural Characterization

### 5.1. XRD Analysis

**Figure 13:**
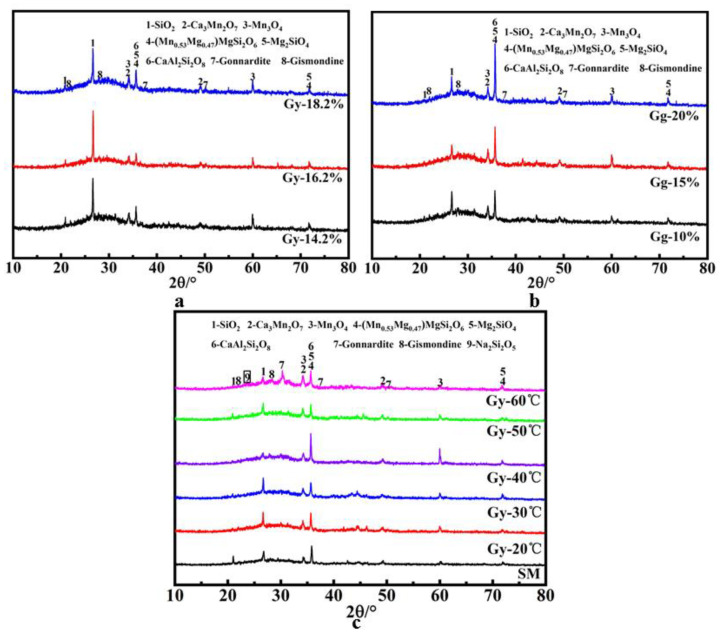
XRD patterns of SMNA upon curing for 28 days: (**a**) solid activator for 28 days; (**b**) liquid activator at 28 days; (**c**) different curing temperatures for 28 days.

As shown in Figure 13a–c, the envelope peak of the amorphous structure can be seen at 20–40° 2θ. This sub-peak is a geopolymer gel characteristic peak [39,40], indicating that the gel generated in aggregate is amorphous. In addition, zeolite and zeolite phases are identified in the aggregate, and the formation of zeolite is related to Ca^2+^ and Na^+^. Compared with the raw material, the quartz peak at 20.8° 2θ is significantly reduced, indicating its dissolution by the alkaline activator in the geopolymerization reaction and conversion into the gel. The presence of pyroxene (Mn_0.53_Mg_0.47_) MgSi_2_O_6_, magnesium olivine (Mg_2_SiO_4_), anorthite (CaAl_2_Si_2_O_8_), Mn_3_O_4_, and Ca_3_Mn_2_O_7_ in the aggregate indicates that these substances do not participate in the geopolymerization reaction.

According to Figure 13c, when the curing temperature is between 20 and 40 °C, no Na_2_Si_2_O_5_ diffraction peak appears in the aggregate, indicating lower water evaporation. The ground polymerization reaction is rapid, and the gel is gradually generated, increasing the aggregate strength. With the increase in the curing temperature to 60 °C, the Na_2_Si_2_O_5_ diffraction peak appears in the aggregate, indicating that the mass evaporation of water in the system at this temperature yields poor aggregate polymerization, still exhibiting some sodium silicate that does not participate in the reaction, yielding lower gel generation and a lower aggregate strength.

### 5.2. SEM Analysis

**Figure 14:**
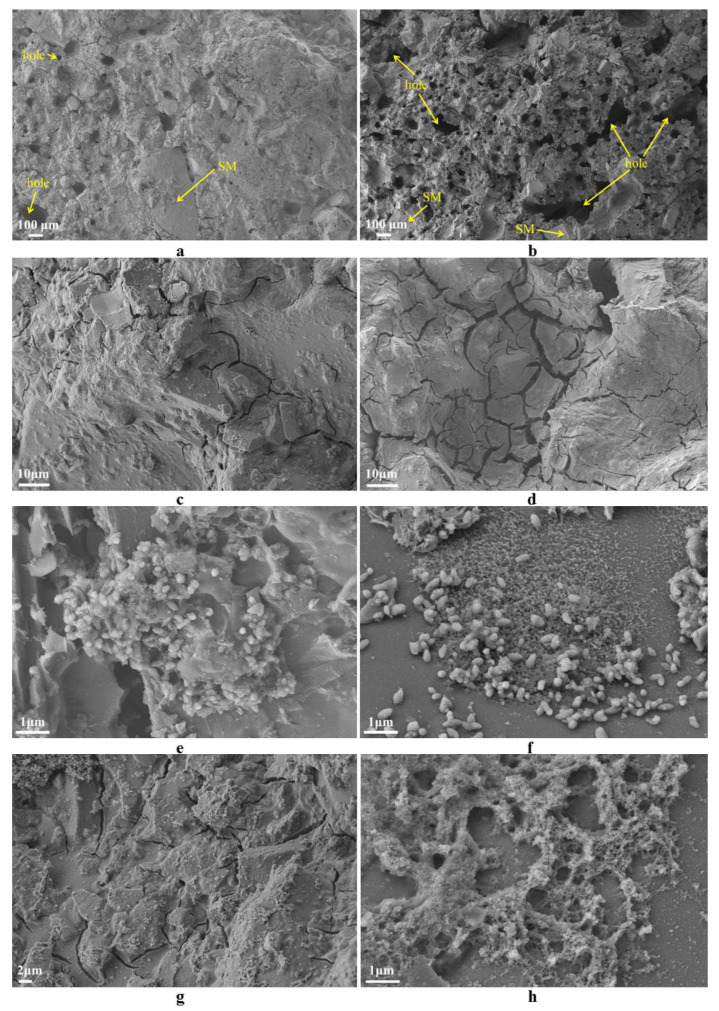
SEM images of SMNA: (**a**) 2–6; (**b**) 2–8; (**c**) 3–4; (**d**) 3–5; (**e**) 2–6; (**f**) 2–6; (**g**) 2–8; (**h**) 2–8.

Figure 14a–d, are SEM images measured at low magnification, where a–b is the aggregate prepared using the liquid activator under curing at 40 ℃, with a few small holes locally distributed in the aggregate section and close stacking between particles. At the same time, the gel material generated from the raw material and alkaline activator is tightly wrapped with the unreacted SM slag particles. However, upon curing at 60 ℃, the aggregate exhibits many large pores, surface cracks, and a loose structure. The c and d panels show the aggregate prepared using the solid activator. It displays a smooth surface, fewer cracks, and compact structure upon curing at 40 ℃. However, cracks are widely distributed upon curing at 60 ℃.

Figure 14e–h, represent the SEM images at high magnification. There are many particles and flock-like products on the surface of the SM slag aggregate due to the gel generated by the reaction of raw materials and alkaline activator. Some particles and flock-like materials are interlaced and closely stacked together, which increases the aggregate strength.

### 5.3. FTIR Analysis

**Figure 15:**
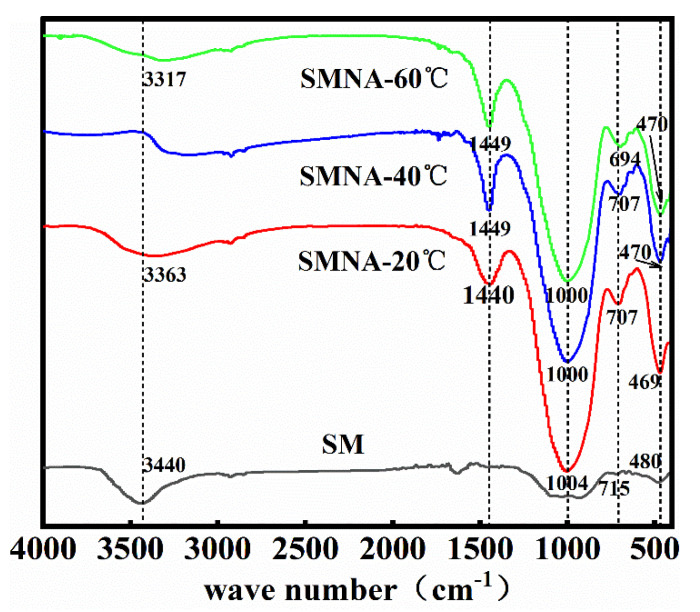
FTIR spectra of SMNA at different curing temperatures.

Figure 15 shows the FTIR spectra of SM slag and SMNA after 28 days in the wavenumber range of 400–4000 cm^−1^. Figure 15 illustrates Si-O-Si bending vibration band between 450 and 650 cm^−1^ [41]. Compared with raw materials, the absorption peak of SM slag aggregate shifts toward lower wavenumbers, decreasing by 10 and 11 cm^−1^, indicating that the Si-O bond length in aggregate increases, while the bond angle decreases, as well as the molecular dynamic constant [42]. The absorption peak at 690–710 cm^−1^ is attributed to Si-O symmetric stretching vibration [43]. The absorption peak at 1000 -1030 cm^−1^ is caused by the asymmetric stretching vibration of tetrahedral TO_4_ (T = Si or Al) in Si-O-T [44]. The peak in this range represents the phase of amorphous silica-aluminate gel. Therefore, it can be indicated that the geopolymerization reaction between SM slag and alkaline initiator occurs. This is consistent with the XRD results of all aggregates, showing wide amorphous peak phases in the 20–40° 2θ range. After adding an alkaline activator, each aggregate sample exhibits an absorption peak at 1400–1450 cm^−1^ originating from the C-O stretching vibration. This indicates that free ions in the aggregate react with CO_2_ and the sample becomes carbonized [45], probably during the experimental testing or in the dryer. The stretching vibration of -OH in the range of 3250–3750 cm^−1^ [46] originates from the presence of structural water and free water. The absorption peaks appear in raw materials and aggregates in this range, indicating a small amount of water in raw materials, while free water in aggregates may be absorbed on the surface or trapped in the pores of reaction products. Structural water exists in the aggregate as a part of hydration products [47].

### 5.4. Leaching Analysis of Heavy Metals in SMNA

ICP-7000 was used to detect and analyze the presence of some heavy metals in the water-quenched SM slag and aggregate, and the results are listed in Table 5 and Table 6.

The aggregate in this experiment is prepared from 100% water-quenched SM slag. Table 5 indicates the presence of some heavy metals: Mn, Cu, Cr, and Ni in the water-quenched SM slag. In this paper, nine groups of aggregate samples were selected to test the heavy metal leaching from the aggregate according to the heavy metal toxic leaching method. The test results are shown in Table 6. The leaching toxicity of Cr, Cu, Ni, and other heavy metals of the prepared aggregate is far lower than the limit given by the standard GB5085.3-2007 Hazardous Waste Identification Standard Leaching Toxicity Identification, indicating that the prepared aggregate does not pose leaching risk and can be applied in the field of building materials. Regarding the Mn leaching results, only the sixth group shows a high Mn content, while it is lower than 0.3210 for the other eight groups. The amount of alkaline activator in the aggregate may affect the immobilization of heavy metals. In general, a higher amount of alkaline activators can reduce the leaching of heavy metals. Therefore, choosing an appropriate alkaline activator dose can effectively cure the aggregate heavy metals and reduce the amount of alkaline activator agent and the final cost.

## 6. Conclusions

In this study, SMNA comprising 100% wt. waste materials (SM and FA), SMNA were produced through the cold bonding method. Through exploring the effects of grinding time, amounts of solid and liquid alkaline activators, curing temperature, and the amount of added fly ash on SMNA properties. Based on the experimental results, the following conclusions can be drawn:

(1) Considering the cost and aggregate properties, the room temperature curing was the optimal curing method.
(2) When the amounts of liquid and solid activators are 16.2% and 15%, respectively, the obtained SMNA prepared with silico-manganese (SM) slag showed good properties. It had bulk densities of 1397.5–1476.9 kg/m^3^, water absorption of 5.38–5.88%, apparent density of 2365.8–2384.7 kg/m^3^, and pellet strength of 24.7–25.6 MPa.
(3) Considering the single-ball strength and cylinder compressive strength of the aggregate, the aggregate performance reached the optimal value when the FA content was 20%. Its bulk density was 1236.6 kg/m^3^, water absorption was 4.90%, apparent density was 1973.12 kg/m^3^, the cylinder compressive strengths after 3 and 28 days were 24.7 MPa and 5.7 MPa, respectively. The addition of FA reduced the bulk density, while the strength was not affected. In the subsequent application, the non-burning lightweight aggregate could be prepared.
(4) By comparing the granulation efficiency of solid and liquid activators, the granulation efficiency of the solid activator was higher, and it was easy to control the aggregate’s particle size.
(5) The results of aggregate toxicity leaching showed that Cu, Cr, Ni, Pb, and Cd in SM slag aggregate could meet the limits of GB 5085.3-2007, without causing any harm to the environment, so they can safely replace the natural aggregate.

## Figures and Tables

**Table 1:** Main chemical constituents of SM and FA (%).

Material	SiO_2_	Al_2_O_3_	CaO	MgO	MnO	Fe_2_O_3_	K_2_O	SO_3_	Na_2_O	Others
SM	42.17	21.66	20.71	5.60	5.77	-	1.08	1.37	-	1.973
FA	44.8	22.6	6.2	1.8	-	5.7	1.7	-	1.5	15.7

**Table 2:** Liquid activator agent preparation SMNA formula table.

Type	SM (%)	Ratio of the Liquid Activator and Raw Material in the Mixture (%)	Curing Temperature (°C)	Curing Time (Day)
1-1	100	16.2	60	3
1-2	100	16.2	60	3
1-3	100	16.2	60	3
1-4	100	16.2	60	3
2-1	100	14.2	25	3; 28
2-2	100	16.2	25	3; 28
2-3	100	18.2	25	3; 28
2-4	100	16.2	20	3; 28
2-5	100	16.2	30	3; 28
2-6	100	16.2	40	3; 28
2-7	100	16.2	50	3; 28
2-8	100	16.2	60	3; 28

Note: the ratio of liquid sodium silicate to NaOH is 5:1, and the amount of water added is controlled at 4.0%.

**Table 3:** Solid activator agent preparation SMNA formula table.

Type	SM (%)	Ratio of the Solid Activator and Raw Material in the Mixture (%)	Curing Temperature (°C)	Curing Time (Day)
3-1	100	10	25	3; 28
3-2	100	15	25	3; 28
3-3	100	20	25	3; 28
3-4	100	15	40	3; 28
3-5	100	15	60	3; 28

Note: the amount of water added should be controlled at 9–10%.

**Table 4:** Fly ash addition amount to prepare SMNA formula table.

Type	SM (%)	FA (%)	Ratio of the Solid Activator and Raw Material in the Mixture (%)	Curing Temperature (°C)	Curing Time (Day)
4-1	90	10	15	25	3; 28
4-2	80	20	15	25	3; 28
4-3	70	30	15	25	3; 28
4-4	60	40	15	25	3; 28
4-5	50	50	15	25	3; 28

Note: The water addition is controlled at 12.8–16.8%, where 1 represents the effect of grinding time on aggregate; 2 represents the effect of liquid activator on aggregate; 3 represents the effect of solid activator on aggregate; 4 represents the influence of the amount of the added FA on aggregate.

**Table 5:** Determination and analysis of heavy metals in SM slag (in mg/kg).

Mn	Pb	Cr	Cd	Co	Ni	Cu
5.18 × 10^4^	≤0.005	28.62	≤0.005	≤0.005	25.30	18.75

**Table 6:** Comparison of the heavy metal leaching toxicity between the aggregate and the standard (in mg/L).

Type	Mn	Cr	Cu	Ni	Pb
2-1	0.2540	≤0.005	≤0.005	≤0.010	≤0.005
2-2	0.1950	≤0.005	≤0.005	≤0.010	≤0.005
2-3	0.1975	≤0.005	≤0.005	≤0.010	≤0.005
3-1	0.1585	≤0.005	≤0.005	≤0.010	≤0.005
3-2	0.0970	≤0.005	≤0.005	≤0.010	≤0.005
3-3	11.27	≤0.005	≤0.005	≤0.010	≤0.005
2-6	0.1855	≤0.005	≤0.005	≤0.010	≤0.005
2-7	0.3210	≤0.005	≤0.005	≤0.010	≤0.005
2-8	0.2815	≤0.005	≤0.005	≤0.010	≤0.005
GB5085.3-2007 (limit value)	-	15	100	5	5

## Data Availability

The data presented in this study are available on request from the authors.

## References

[1] Dong B.Q., Luo X.L., Tian K.G., Hong S.X., Wang Y.S. (2021). Preparation and characterization of alkali-activated lithium slag-based artificial aggregates. Mater. Rev..

[2] Risdanareni P., Schollbach K., Wang J.Y., Belie N.D. (2020). The effect of NaOH concentration on the mechanical and physical properties of alkali activated fly ash-based artificial lightweight aggregate. Constr. Build. Mater..

[3] Shahane H.A., Patel S. (2020). Influence of curing method on characteristics of environment-friendly angular shaped cold bonded fly ash aggregates. J. Build. Eng..

[4] Gu X., Li X., Zhang W., Gao Y., Kong Y., Liu J., Zhang X. (2021). Effects of HPMC on workability and mechanical properties of concrete using iron tailings as aggregates. Materials.

[5] Abdullah A., Hussin K., Abdullah M., Yahya Z., Sochacki W., Razak R.A., Bloch K., Fansuri H. (2021). The Effects of various concentrations of NaOH on the inter-particle gelation of a fly ash geopolymer aggregate. Materials.

[6] Jeon D., Yum W.S., Song H., Yoon S., Bae Y., Oh J.E. (2020). Use of coal bottom ash and CaO-CaCl_2_-activated GGBFS binder in the manufacturing of artificial fine aggregates through cold-bonded pelletization. Materials.

[7] Mo L., Yang S., Huang B., Xu L., Feng S., Deng M. (2020). Preparation, microstructure and property of carbonated artificial steel slag aggregate used in concrete. Cem. Concr. Compos..

[8] Li S.C., Zhu C.J., Ning W. In Study on the process of producing high quality rock wool by using thermal silicon manganese slag based on pool kiln process. Proceedings of the National Glass Furnace Technology Symposium, National Glass Furnace Technology Seminar and Exchange Conference.

[9] Dou L.R. (2017). Ultilization of silicomanganese slag in building materials. China Manganese Ind..

[10] Huang T.Z. (2012). Study on the Utilization of SiMn Slag in Construction Materials. Master’s Thesis.

[11] Patil A.V., Pande A.M. (2011). Behaviour of silico manganese slag manufactured aggregate as material for road and rail track construction. Adv. Mater. Res..

[12] Li W.B., Yu W.G., Tian B.S. (2003). Production of ordinary portland cement from silico-manganese slag. China Cem..

[13] Allahverdi A., Ahmadnezhad S. (2014). Mechanical activation of silicomanganese slag and its influence on the properties of portland slag cement. Powder Technol..

[14] Zhang D.Y., Ju B.X., Li P., Zhan L., Zhang C., Zhang J.L., Dai L.W., He Z.Y. (2012). Experimental study of compound admixture mixed with siliconmanganese slag. Coal Ash.

[15] Frías M., Rojas M.I.S.D., Rodríguez C. (2009). The influence of SiMn slag on chemical resistance of blended cement pastes. Constr. Build. Mater..

[16] Ting M.Z.Y., Wong K.S., Rahman M.E., Joo M.S. (2020). Mechanical and durability performance of marine sand and seawater concrete incorporating silicomanganese slag as coarse aggregate. Constr. Build. Mater..

[17] Colangelo F., Messina F., Cioffi R. (2015). Recycling of MSWI fly ash by means of cementitious double step cold bonding pelletization: Technological assessment for the production of lightweight artificial aggregates. J. Hazard. Mater..

[18] Tajra F., Elrahman M.A., Lehmann C., Stephan D. (2019). Properties of lightweight concrete made with core-shell structured lightweight aggregate. Constr. Build. Mater..

[19] Tang P., Xuan D., Cheng H.W., Poon C.S., Tsang D.C.W. (2020). Use of CO_2_ curing to enhance the properties of cold bonded lightweight aggregates (CBLAs) produced with concrete slurry waste (CSW) and fine incineration bottom ash (IBA). J. Hazard. Mater..

[20] Tang P., Brouwers H.J.H. (2017). Integral recycling of municipal solid waste incineration (MSWI) bottom ash fines (0–2 mm) and industrial powder wastes by cold-bonding pelletization. Waste Manage..

[21] Tian K.G., Wang Y.S., Hong S.X., Zhang J.R., Hou D.S., Dong B.Q., Xing F. (2021). Alkali-activated artificial aggregates fabricated by red mud and fly ash: Performance and microstructure. Constr. Build. Mater..

[22] Tang P., Xuan D., Li J., Cheng H.W., Poon C.S., Tsang D.C.W. (2020). Investigation of cold bonded lightweight aggregates produced with incineration sewage sludge ash (ISSA) and cementitious waste. J. Clean. Prod..

[23] Gómez M., Peisino L.E., Kreiker J., Gaggino R., Cappelletti A.L., Martín S.E., Uberman P.M., Positieri M., Raggiotti B.B. (2020). Stabilization of hazardous compounds from WEEE plastic: Development of a novel core-shell recycled plastic aggregate for use in building materials. Constr. Build. Mater..

[24] Geetha S., Ramamurthy K. (2010). Environmental friendly technology of cold-bonded bottom ash aggregate manufacture through chemical activation. J. Clean. Prod..

[25] Narattha C., Chaipanich A. (2018). Phase characterizations, physical properties and strength of environment-friendly cold-bonded fly ash lightweight aggregates. J. Clean. Prod..

[26] Shang X., Li J., Zhan B. (2020). Properties of sustainable cellular concrete prepared with environment-friendly capsule aggregates. J. Clean. Prod..

[27] Tuncel E.Y., Pekmezci B.Y. (2018). A sustainable cold bonded lightweight PCM aggregate production: Its effects on concrete properties. Constr. Build. Mater..

[28] Alqahtani F.K., Rashid K., Zafar I., Iqbal Khan M. (2021). Assessment of morphological characteristics and physico-mechanical properties of geopolymer green foam lightweight aggregate formulated by microwave irradiation. J. Build. Eng..

[29] Manikandan R., Ramamurthy K. (2008). Effect of curing method on characteristics of cold bonded fly ash aggregates. Cem. Concr. Compos..

[30] Vasugi V., Ramamurthy K. (2014). Identification of design parameters influencing manufacture and properties of cold-bonded pond ash aggregate. Mater. Des..

[31] Zhu W.X., Feng L., Zhou H.M., Qin Y.C., Luan H.X. (2017). Analysis on the development and application of a new type of ash haydite. Concrete.

[32] Wang K.T. (2016). Reaction Mechanism and Applications of ALKALI-BASED Geopolymers At Low Temperature and Vacuum Conditions. Ph.D. Thesis.

[33] Cheng T.W., Chiu J.P. (2003). Fire-resistant geopolymer produced by granulated blast furnace slag. Miner. Eng..

[34] Nazari A., Bagheri A., Riahi S. (2011). Properties of geopolymer with seeded fly ash and rice husk bark ash. Mater. Sci. Eng. A.

[35] Zhang H.B., Zheng Z.G., Kou J.L. (2017). Mechanical properties of silica fume-aluminum oxide geopolymer. J. Build. Mater..

[36] Baykal G., Dven A.G. (2000). Utilization of fly ash by pelletization process; theory, application areas and research results. Resour. Conserv. Recycl..

[37] Elisabeth V., Tran L.H., Pasquier L.C., Blais J.F., Mercier G. (2021). Valorization of apatite mining flotation residues by the manufacture of artificial aggregates. Resour., Conserv. Recycl..

[38] Manikandan R., Ramamurthy K. (2007). Influence of fineness of fly ash on the aggregate pelletization process. Cem. Concr. Compos..

[39] Chen Z., Li J.S., Zhan B.J., Sharma U., Poon C.S. (2018). Compressive strength and microstructural properties of dry-mixed geopolymer pastes synthesized from GGBS and sewage sludge ash. Constr. Build. Mater..

[40] Yaseri S., Hajiaghaei G., Mohammadi F., Mahdikhani M., Farokhzad R. (2017). The role of synthesis parameters on the workability, setting and strength properties of binary binder based geopolymer paste. Constr. Build. Mater..

[41] Lodeiro I.G., Macphee D.E., Palomo A., Fernández-Jiménez A. (2009). Effect of alkalis on fresh C–S–H gels. FTIR analysis. Cem. Concr. Compos..

[42] Sha D., Pan B.F., Li Y.C., Wang B.M. (2021). Studies on preparation and performance of alkali-activated coal-based synthetic natural gas slag geopolymer. China J. Highw. Transp..

[43] Liu X., Jiang J., Zhang H., Li M., Zhang Z. (2020). Thermal stability and microstructure of metakaolin-based geopolymer blended with rice husk ash. Appl. Clay Sci..

[44] Aiken T.A., Kwasny J., Wei S., Soutsos M.N. (2018). Effect of slag content and activator dosage on the resistance of fly ash geopolymer binders to sulfuric acid attack. Cem. Concr. Res..

[45] Chen K., Lin W.T., Liu W. (2021). Effect of NaOH concentration on properties and microstructure of a novel reactive ultra-fine fly ash geopolymer. Adv. Powder Technol..

[46] Yu Q.Q., Li S.L., Li H., Chai X.N., Bi X.Y., Liu J.L., Ohnuki T. (2019). Synthesis and characterization of Mn-slag based geopolymer for immobilization of Co. J. Clean. Prod..

[47] Sha D., Pan B.F., Sun Y.R. (2020). Investigation on mechanical properties and microstructure of coal-based synthetic natural gas slag (CSNGS) geopolymer. Constr. Build. Mater..

